# General practitioners' conceptions about treatment of depression and factors that may influence their practice in this area. A postal survey

**DOI:** 10.1186/1471-2296-6-21

**Published:** 2005-05-16

**Authors:** Stig J Andersson, Margareta Troein, Gunnar Lindberg

**Affiliations:** 1 Lund University, Department of clinical Sciences Malmö, Family Medicine, MAS, S-20502 Malmö, Sweden; 2 The NEPI Foundation, Malmö, Sweden; 3 The Research department of primary care, County Council of Värmland, Karlstad, Sweden

## Abstract

**Background:**

The way GPs work does not appear to be adapted to the needs of depressive patients. Therefore we wanted to examine Swedish GPs' conceptions of depressive disorders and their treatment and GPs' ideas of factors that may influence their manner of work with depressive patients.

**Methods:**

A postal questionnaire to a stratified sample of 617 Swedish GPs.

**Results:**

Most respondents assumed antidepressive drugs effective and did not assume that psychotherapy can replace drugs in depression treatment though many of them looked at psychotherapy as an essential complement. Nearly all respondents thought that clinical experiences had great importance in decision situations, but patients' own preferences and official clinical guidelines were also regarded as essential. As influences on their work, almost all surveyed GPs regarded experiences from general practice very important, and a majority also emphasised experiences from private life. Courses arranged by pharmaceutical companies were seen as essential sources of knowledge. A majority thought that psychiatrists did not provide sufficient help, while most respondents perceived they were well backed up by colleagues.

**Conclusion:**

GPs tend to emphasize experiences, both from clinical work and private life, and overlook influences of collegial dealings and ongoing CME as well as the effects of the pharmaceutical companies' marketing activities. Many GPs appear to need more evidence based knowledge about depressive disorders. Interventions to improve depression management have to be supporting and interactive, and should be combined with organisational reforms to improve co-operation with psychiatrists.

## Background

Mood disturbances are widespread in western societies and general practitioners (GPs) see many patients with depressive disorders[1,2]. Only a minority of these patients consult directly for psychological problems. More often they consult their doctors because of bodily symptoms[3]. In addition, depressive disorders often present combined with anxiety states[4,5] and a large proportion of the depressed patients in primary care also suffer from concurrent somatic diseases[6]. Consequently, when GPs see depressed patients, they often have to deal with complicated cases. The ways GPs handle these patients have been criticised because of deficient identification and insufficient treatment of the depressive disorders [7-11].

In two previous studies we explored Swedish GPs' conceptions of depressive disorders and their ideas on how their working methods with depressed patients had been shaped. We found a considerable variation in their conceptions about depression, but a relative consensus concerning drug treatment of major depression. As regards manner of working, many interviewed GPs considered individual experiences from general practice and private life as more influential than academic education and professional literature[12,13].

When further examining the geographic variation of sales levels of antidepressant agents (ADs) we found weak but statistically significant associations with some of local GPs' conceptions of factors that may have formed the way they treat depressive patients[14]. Thus, high sales levels correlated positively with a high evaluation of ADs' effectiveness in depression and panic disorders and were inversely correlated with the degree of appreciation of psychotherapy-based treatments. High sales levels were also associated with a high evaluation of GPs' own clinical and private experience, with a positive appreciation of the work with depressed patients and with a high level of participation in the pharmaceutical companies' activities.

The purpose of the present study was to elaborate further the frequencies of Swedish GPs' conceptions of depressive disorders and its treatment and of their ideas of factors that may influence their manner of work with depressive patients.

## Methods

### Participants

The present study made use of the same data collection as the previous study of the relations between GPs' conceptions of treatment of depression and local sales levels of ADs[14]. For that purpose we had surveyed a stratified sampling of GPs based on the population's purchases of ADs in each GP's working area. Information on purchases of ADs for the year 2000 in each Swedish municipality was obtained from Apoteket AB (The National Corporation of Swedish Pharmacies). We selected three groups of municipalities; those with the highest, the average, and the lowest AD sales rates. For each sales group, municipalities were included according to their sales figures until the total number of GPs working in the municipalities of the group approximated 200. In that way we selected 617 GPs working in a total of 60 municipalities. The respondents of a pilot study were not included. The names and addresses were received from *Läkarmatrikeln 2001*, a national register of Swedish physicians.

### Instrument

A postal questionnaire was designed based on the findings in the interview study[12,13]. A five-point Likert scale ranging from "Agree not at all" to "Agree completely" and from "Very little importance" to "Very great importance" was used for most items. The questionnaire was tested in 20 GPs. That pilot study had a response rate of 75 per cent, no internal drop off, i.e. all responders answered all items, and the answers were spread over the alternatives of the Likert scale on most items. After a few modifications the questionnaire was finalized[15].

In the graphs, the GP's answers are combined to a three-point scale after merging alternatives 1 and 2 and alternatives 4 and 5 of the original five-point Likert scale.

### Performance

The survey was carried out in spring 2001. The questionnaires, including a return envelope, were mailed from the NEPI Foundation, a non-profit organisation for studies on epidemiology of drugs, to all the 617 GPs working in the selected municipalities. No incentive for participation was offered. Two reminders were sent if no answer was received, the second of which included a new questionnaire.

After the data collection, the work places of non-respondents were contacted to ascertain whether or not these GPs were still employed at the time of the survey.

The present analysis of data was not reported to the Ethics Committee because our earlier studies had been approved on the administrative level alone.

## Results

After the second reminder 317 of the 617 mailed questionnaires were returned. Among non-respondents 82 had left their work places leaving 535 GPs eligible for the survey. The response rate of the eligible participants was 59.4 percent: 56 percent among the 339 men and 65 percent among the 196 women.

Among respondents, the average age was 48.7 years and they had worked as GPs for 12.7 years on average. Sixty percent were male. Among non-respondents available for the survey, the average age was 59.0 years and 69 percent were male.

To work with depressive patients was perceived a more positive than negative task by 69 per cent of the respondents, "neither positive, nor negative" by 27 per cent and mainly negative by four per cent of the respondents.

**Figure 1:**
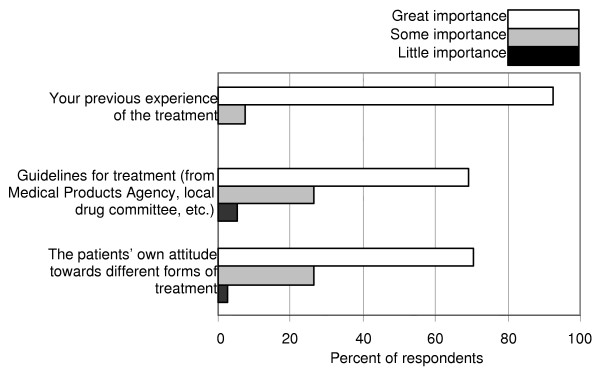
**Factors of importance for decision about antidepressive treatment. **Mark which importance you attach to each of the mentioned factors when you decide about antidepressive treatment: a. The patient's own attitude towards different forms of treatment; b. Guidelines for treatment; c. Your previous clinical experience of the treatment.

When deciding treatment for the depressive patient, nearly all GPs considered that their own clinical experiences of the treatment had great importance, and, for a majority, also patients' own preferences and clinical guidelines to treatment were of major value (Fig. 1).

**Figure 2:**
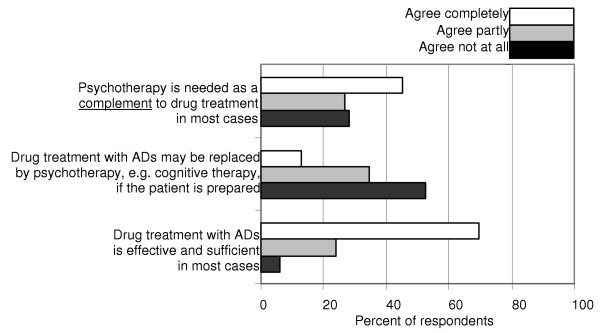
**Psychotherapy and drug treatment. **Give for each statement your degree of agreement: a. Psychotherapy is needed as a compliment to drug treatment in most cases. b. Drug treatment with antidepressive agents (ADs) may be replaced by psychotherapy, e.g. cognitive therapy, if the patient is prepared. c. Drug treatment with ADs is effective and sufficient in most cases.

Treatment of moderate depression includes mainly antidepressant medication and psychotherapy. A majority of the respondents agreed that medication with ADs is effective, and did not assume that psychotherapy can replace drug treatment. A majority agreed, fully or partially, that psychotherapy is needed as a complement to drug treatment (Fig. 2).

**Figure 3:**
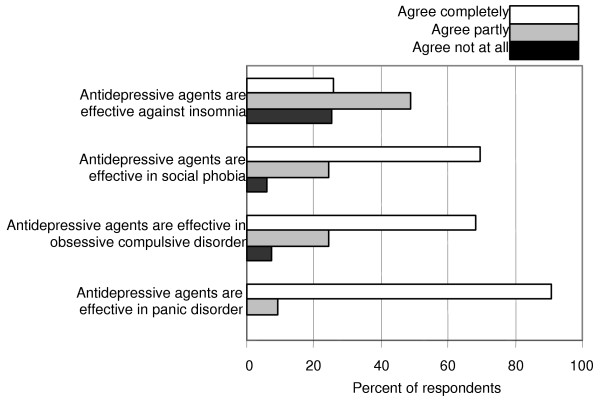
**Use of antidepressive agents in other conditions than depression. **Antidepressive agents can be used in other conditions than depression. Give for each statement your degree of agreement: a. ADs are effective in panic disorder. b. ADs are effective in obsessive-compulsive disorder. c. ADs are effective in social phobia. d. ADs are effective against insomnia.

Most responders had great confidence in ADs for treatment of panic disorder and thought they are effective also in obsessive-compulsive disorders and social phobias (Fig. 3), but the majority of the responders showed a limited reliance on ADs' effectiveness to treat sleep disorders, psychosomatic illness, chronic pain, and premenstrual syndrome.

**Figure 4:**
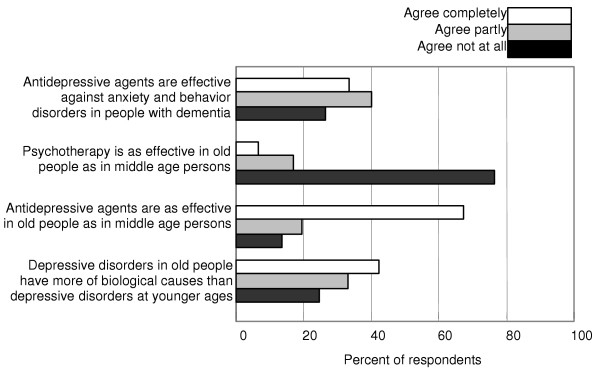
**Depressive disorders in old people. **Here follow some statements on depressive disorders and their treatment in old people. Mark for each statement which degree of agreement you have: a. Depressive disorders in old people have more of biological causes then depressive disorders in younger ages. b. ADs are as effective in old people as in middle age people. c. Psychotherapy is as effective in old people as in middle age persons. d. ADs are effective against anxiety and behaviour disorder in people with dementia.

Two thirds of the responding GPs considered ADs as effective for the treatment of old people as for people at younger ages. Their confidence in psychotherapy for old patients was low. The responders' judgments on the statement that ADs are effective for the treatment of anxiety and behavioural disturbances in patients with dementia were distributed evenly from agree not at all to completely agreement (Fig. 4).

Table 1 displays factors, which, according to the GPs, may have influenced their manner of working with depressed patients. Almost all regarded individual experiences from family medicine as very important, and a large majority also regarded experiences from private life to be of significant importance.

**Table 1:** Factors that may have influenced on GPs' manner of working with depressed patients. Share of respondents that have marked "great" or "very great" importance on the factor in question.

Experiences from general practice	96 %
Experiences from private life	83 %
Residency in psychiatry	57 %
Professional reading	50 %
Education on communication	50 %
A mentor colleague	26 %
Reading fiction literature	20 %
Religious faith	17 %
University education	12 %
Popular reading on psychiatry/psychology	10 %

**Figure 5:**
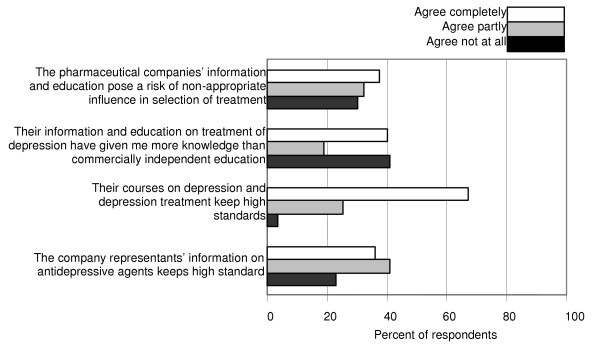
**Pharmaceutical companies' information and courses on depression. **Here follow five statements about the pharmaceutical companies' information and courses on depression and antidepressive agents. Mark for each statement your degree of agreement: a. The company representants' information on antidepressive agents keeps high standards. b. Their courses on depression and depression treatment keep high standards. c. Their information and education on treatment of depression have given me more knowledge than commercially independent education. d. The pharmaceutical companies' information  and education pose a risk of non-appropriate influence in the selection of treatment.

Some issues concerned continuing medical education (CME). Much the same number of GPs considered non-commercial courses to be of great importance as courses arranged by pharmaceutical companies. The respondents regularly took part in more lectures arranged by drug companies than in lectures without involvement of commercial interests (Table 2). Commercial courses were generally regarded as meeting high standards. More than one third of the respondents thought they had received more knowledge from such courses than from commercially independent education. A majority of the respondents agreed partly or completely that commercially sponsored courses may influence physicians in an inappropriate way, but many GPs did not recognise that risk (Fig. 5).

**Table 2:** Respondents' reports of occasions of participation in education/lectures each year. Number of respondents.

	Not regularly	1 – 3 /year	4 – 7/year	>7/year
Arrangements without industry involvement	74	127	71	43
Arranged by pharmaceutical industries	30	141	81	62

Most respondents stated that they had good possibilities to discuss with their colleague GPs. In contrast, a majority claimed that psychiatrists do not provide them with the necessary support for patient care.

## Discussion

Our purpose was to investigate conceptions concerning depressive disorders among Swedish GPs. We found that GPs regarded experiences from family medicine and from private life more important than university and post-graduate education as influences on their work with depressive patients. Most GPs had great confidence in ADs for treatment of depressive disorders and panic disorder and thought that the drugs are effective also in obsessive-compulsive disorders and social phobias. A majority of GPs also agree that commercially sponsored courses may influence physicians in an inappropriate way, but many of them do not recognise that risk.

### Method considerations

For the purpose of an additional study utilizing the same data collection, the sampling procedure was not randomised but stratified. The participants were all GPs who worked in the selected municipalities. They constituted groups of GPs with different levels of prescribing ADs and were from all parts of Sweden. We assume the procedure resulted in an appropriate sample of Swedish GPs.

A response rate of 59 percent among eligible GPs is well in line with comparable surveys among physicians, although such a rate means that the possible impact of selection bias must be considered [16-20]. However, there are solid arguments that the result of the questionnaire can be viewed as representative even if the response rate is moderate, when a homogenous professional group is surveyed on issues of central professional importance [22-24]. We assume that the results are representative of all the selected GPs even if there were minor differences in gender distribution and age between responders and non-responders.

The questionnaire was not validated against any earlier instrument, but the items of the questionnaire were extracted from findings of the previous interview studies among GPs, a procedure that should ensure the relevance of the items [12,13]. The questionnaire was tested by a pilot study that had a response rate of 75 per cent and an extensive spread of responses over the alternatives of the Likert scales. In the present study there were a few internal drop-offs, i.e. some responders did not answer all items. Altogether, we claim that the present study has acceptable representativeness and validity.

### Interpretation of the findings

The respondents opinions of the usefulness of ADs in treatment of moderate depression and anxiety disorders were in accordance with findings in our previous interview study and with actual recommendations[12,25,26]. Psychotherapy was considered most often as a complement to drugs in the treatment of depression. In the previous interview study, the majority of the interviewees presented a positive attitude to psychotherapy[12]. There is now good evidence for the effectiveness of several forms of psychotherapy against moderate depressions [27-34]. These findings appear not to have become generally accepted by Swedish GPs, probably partly due to the limited access to psychotherapists.

A clear majority of the GPs in the present study perceived the work with depressed patients in a predominantly positive way. A notable finding was the great importance they attached to their own experiences from family medicine and from private life. That was valid for both treatment decisions and for their manner of working, and the same came out in the interview study[13]. Judging from these responses, GPs try to consider treatment guidelines as well as patients' attitudes to treatment and their individual experiences of similar situations when they propose treatments. In such complex decision situations, the physicians seem to perceive their individual experiences as indispensable. That is in accordance with Schön, who reported that practitioners gather repertoires of understandings and actions from their experiences, and that they use their repertoires to guide their professional actions. The quality of this guidance varies. A practitioner must have worked through his/her experiences by discussions and reflection if his/her repertoire should serve as a valuable guide[35].

To a substantial degree, the outcome in drug treatment of depression is dependent on the therapeutic alliance between the physician and the patient[36]. This alliance is built up by the physician's ability to communicate, which is acquired mainly through individual experience, guiding and reflective reading.

When professionals rely primarily on personal experiences as grounds for clinical assessment and intervention, they demonstrate preference for "knowledge in action" over inquiring scientific knowledge, and, tentatively, a scepticism regarding the usefulness of scientific literature[37]. Many respondents' low or moderate evaluation of their undergraduate education indicates such scepticism. Lennarson Greer writes about "the problem of uncertainty" as a key problem of the medical profession[38]. For many physicians, trust in personal experiences and collegial support appear to be better ways to handle the worry of uncertainty than reading scientific literature[38,39].

Thus, the respondents' emphasis on their own experiences is understandable, but, on the other side, a low evaluation of scientific and evidence-based knowledge may imply insufficient capability to form an accurate opinion of a patient's state and to propose an optimal treatment.

Even if the respondents ranked their own experiences of clinical work and private life as most important, they also indicated that lectures and professional reading are important sources of knowledge about depression. Further, the striking increase of prescriptions of ADs in the 1990s indicates that recognition and management of depressive disorders have changed[40,41]. Reasonably, that is due to other influences than GPs' own experience. Psychiatric specialists have probably had an impact on GPs' prescribing, partly directly by initiating or recommending treatment for patients, partly indirectly by education and informal consultations. The pharmaceutical companies' marketing of ADs is another powerful incentive to increase the use of ADs. In our previous study we learned that high sales levels correlated with more participation in the pharmaceutical companies' activities[14]. Also other studies have documented the impact of commercial marketing on prescribing and professional behaviour of physicians [42-45].

GPs stand out as a cultural subgroup, where ongoing educational, collegial and marketing influences are obvious from outside but not quite clear for the members themselves. Many GPs appear to overlook these external influences on their thinking and professional behaviour, while they tend to over-emphasize individual experiences as a base of their clinical work.

To some extent, GPs' disappointment with psychiatric services may depend on unrealistic ideas about psychiatrists' ability to solve GPs' problems. It has been pointed out that GPs and psychiatrists have different preconditions in their clinical work, and that they meet different kinds of depressed patients[3,46]. Projects to improve depression management have reported that simple guideline implementation and educational strategies were generally ineffective. In short term evaluation, effective strategies were those with broad interventions that incorporated clinical education, enhanced team work and a greater integration between primary care and psychiatry [46-50].

## Conclusion

In their conceptions of factors that form their way to treat depressed patients, GPs tend to emphasize experiences, both from clinical work and private life, and overlook influences of collegial dealings and ongoing CME as well as the effects of the pharmaceutical companies' marketing activities. Many GPs appear to need more evidence based knowledge about depressive disorders. Interventions to improve depression management have to be supporting and interactive and combined with organisational reforms to improve co-operation with psychiatrists.

## Competing interests

The author(s) declare that they have no competing interests.

## Authors' contributions

The authors have planned and carried out the study together. SJA wrote the first draft of the manuscript. GL organised data and constructed the graphs. MT was active in planning the study, organising the data and the linguistic formulation of the article. The authors revised the manuscript together.

## Pre-publication history

The pre-publication history for this paper can be accessed here:


